# From Dysbiosis to Healthy Skin: Major Contributions of *Cutibacterium acnes* to Skin Homeostasis

**DOI:** 10.3390/microorganisms9030628

**Published:** 2021-03-18

**Authors:** Miquel Rozas, Astrid Hart de Ruijter, Maria Jose Fabrega, Amine Zorgani, Marc Guell, Bernhard Paetzold, Francois Brillet

**Affiliations:** 1 S-Biomedic, JLABS, Turnhoutseweg 30, 2340 Beerse, Belgium; miquel@sbiomedic.com (M.R.); astrid@sbiomedic.com (A.H.d.R.); amine@sbiomedic.com (A.Z.); marc.guell@upf.edu (M.G.); bernhard.paetzold@sbiomedic.com (B.P.); 2 Department of Experimental and Health Sciences, Universitat Pompeu Fabra (UPF), C. Dr. Aiguader 88, 08003 Barcelona, Spain; maria-jose.fabrega@upf.edu

**Keywords:** skin microbiota, microbiome dysbiosis, skin disorders, *Cutibacterium acnes*, topical bacteriotherapy

## Abstract

*Cutibacterium acnes* is the most abundant bacterium living in human, healthy and sebum-rich skin sites, such as the face and the back. This bacterium is adapted to this specific environment and therefore could have a major role in local skin homeostasis. To assess the role of this bacterium in healthy skin, this review focused on (i) the abundance of *C. acnes* in the skin microbiome of healthy skin and skin disorders, (ii) its major contributions to human skin health, and (iii) skin commensals used as probiotics to alleviate skin disorders. The loss of *C. acnes* relative abundance and/or clonal diversity is frequently associated with skin disorders such as acne, atopic dermatitis, rosacea, and psoriasis. *C. acnes*, and the diversity of its clonal population, contributes actively to the normal biophysiological skin functions through, for example, lipid modulation, niche competition and oxidative stress mitigation. Compared to gut probiotics, limited dermatological studies have investigated skin probiotics with skin commensal strains, highlighting their unexplored potential.

## 1. Cutibacterium and *Cutibacterium acnes* in the Skin Microbiome

**Figure 1:**
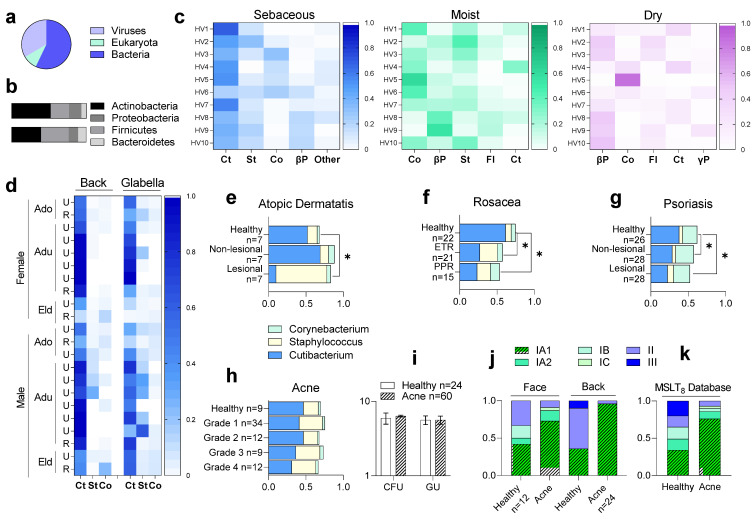
*Cutibacterium acnes* in the human skin microbiome. (**a**) Relative abundance of skin microbiota across kingdoms in 15 heathy volunteers (HV) (9 males, 6 females) sampled in 18 different skin sites, adapted from Oh et al., 2014 [7]. Bacterial genomes predominate at most sites. (**b**) The analysis of 16S ribosomal RNA of 10 HV (20 sites sampled, upper bar graph) and 9 HV (27 sites sampled, lower bar graph) shows that most of the sequences are attributed to four phyla, adapted from Grice et al., 2009 [13] and Costello et al., 2009 [14], respectively. Over this, skin microenvironments also vary drastically in their level of bacterial diversity. (**c**) In 10 HV sampled in sebaceous, moist, and dry sites, the relative abundance of Cutibacterium (Ct, formerly Propionibacterium), Staphylococcus (St), Corynebacterium (Co), Betaproteobacteria (βP), Flavobacteria (Fl), and Gammaproteobacteria (γP) is strongly shaped by the niche, adapted from Grice et al., 2009 [13]. (**d**) The predominating relative abundance of Cutibacterium in sebaceous sites is relatively stable over the 22 HV from different genders, ages (Ado: teenager, Adu: adult, Eld: elderly), and locations (U: urban, R: rural), adapted from Findley et al., 2013 [17] and Ying et al., 2015 [18]. However, this relative abundance of Cutibacterium genus is significantly decreased (*: *p*-value < 0.05) in (**e**) atopic dermatitis lesional regions (2 sebaceous (interscapular and retroauricular) and 1 moist (antecubital fossa) sites sampled in 18 to 60yo males (*n*: number of volunteers)), adapted from Francuzik et al., 2018 [29], (**f**) rosacea lesional regions (ETR: erythematotelangiectatic rosacea, PPR: papulopustular rosacea, 1 sebaceous site (cheek) sampled in 18 to 64yo males and females), adapted from Wang et al., 2020 [39], and (**g**) psoriasis non-lesional and lesional regions sampled in 1 sebaceous (scalp), 2 moist (axilla and gluteal), and 3 dry (trunk, arm, leg) skin sites, adapted from Chang et al., 2018 [47]. (**h**) Interestingly, no significant alteration of the relative abundance of Cutibacterium genus was observed over 67 patients (22-23 year old) with no and/or different acne severity sampled on the cheek, adapted from Li et al., 2019 [61]. (**i**) No difference in the bacterial load (log colony forming units (CFU) and log genomic units (GU) per strip) between 15-30yo male and female healthy or acne patients sampled on the face, adapted from Pecastaings et al., 2018 [62]. The sharp difference between healthy and acne patients is in the decrease in the diversity of *C. acnes* phylotypes. (**j**) Comparison of the relative abundance of the 6 phylotypes of *C. acnes* between 16-35yo male and female healthy and acne patients, adapted from Dagnelie et al., 2017 [65]. (**k**) Relative abundance and statistically significant enrichment of type IA1 with acne is confirmed by the analysis of the current MSLT_8_ isolate database, adapted from McLaughlin et al., 2019 [64].

The skin is the largest (2 m^2^) and most visible organ of the human body [1]. Constituted by three immunologically active layers [2]—the epidermis (75 to 150 µm), the dermis (<2 mm) and the hypodermis (1−2 mm) [3]—the skin is also the largest epithelial surface for interaction with microbes [4]. The outer layer (the stratum corneum), the appendages (sweat and sebaceous glands, hair follicles) [5], and the subepidermal compartments [6] are indeed colonized by a myriad of microorganisms, including, on the skin’s surface, eukaryotes (10%), viruses (30%), and bacteria (60%) (Figure 1A) [7]. Micro-eukaryotes inhabiting human healthy skin are mainly fungi, mostly overrepresented by the genus Malassezia, followed by Penicillium and Aspergillus [8,9]. The skin virome is composed of both human and bacterial viruses. Cutaneous β and γ human papillomaviruses are commonly present on the superficial layers of the skin in most individuals but are very scarcely represented compared to the phages of the bacterial communities living on the skin [10,11]. Within the dominant bacterial kingdom, representing a total of 10^10^ individuals on the skin [12], four major phyla compose the microbial communities: Actinobacteria (36–51%), Firmicutes (24–34%), Proteobacteria (16–11%), and Bacteroidetes (6–9%) (Figure 1B) [13,14]. However, among the different types of skin, these proportions are strongly shaped by the skin’s physiological characteristics, such as temperature, pH, UV light exposure, moisture/humidity, and sebum content [15]. Thus, three major topographical categories provide specific environmental niches: the dry sites (e.g., volar forearm and palm) colonized by a majority of Betaproteobacteria, the moist/humid areas (e.g., bend of elbow) predominated by the genera Staphylococcus and Corynebacterium, and the oily/sebaceous sites (e.g., face and back) largely predominated by the genus Propionibacterium, followed by Staphylococcus and Corynebacterium (Figure 1C) [16]. In these lipid-rich microenvironments, although not exclusively, the relative abundance of bacterial populations is influenced by gender, age, and geographical origin. Nonetheless, Propionibacterium still predominates, with a relative stability in the sebaceous sites of healthy individuals (Figure 1D) [17,18,19]. It cohabitates with the other bacterial populations but also with the viruses (e.g., bacteriophages), fungi (e.g., Malassezia), and parasites (e.g., Demodex) colonizing specific environments—for example, the human hair follicle [20]. This genus, recently reclassified and renamed Cutibacterium [21], is a sentinel of the healthy human skin microbiome [22]. Alteration of its structure is associated with dysbiosis and disease, including atopic dermatitis (AD), psoriasis, rosacea, and acne [23,24]. Systemic antibiotics targeted at reducing *C. acnes* can also disrupt the equilibrium, which may lead to opportunistic infections in the hair follicle by competing species (e.g., *Pseudomonas* species) [25].

**Figure 2:**
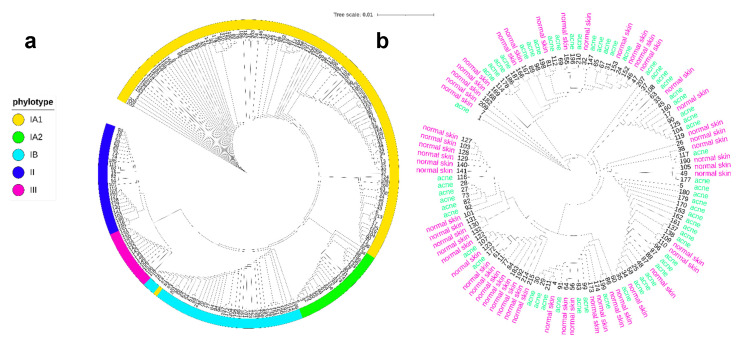
*Cutibacterium acnes* clonal population structure and distribution on normal and acne skin. (**a**) Neighbor-joining tree created using iTOL tool [66] from concatenated nucleotide sequences of MLST schemes [67] from all the current strains in the database published by Jolley et al., 2018 [68]. Over the 215 different isolates, 114 (53%) are classified in the subtype IA_1_, 22 (10%) in the subtype IA_2_, and 39 (18%) in the subtype IB of the major phylogenetic group type I. The rest are classified in the phylogenetic groups II (26 isolates, 12%) and III (14 isolates, 6%). Considering the sources of all the strains in the database, the majority, 112 (52%), have been isolated from skin samples. The rest of the strains have been isolated from samples categorized as eye, prostate, other, blood, soft tissue, medical device, spinal disc, dental, and bone (19, 18, 14, 13, 12, 10, 10, 6, and 1%, respectively). (**b**) Neighbor-joining tree from concatenated nucleotide sequences of MLST schemes from all the isolates sourced as skin samples (acne and normal skin). From the total of 112 strains, 59 have been isolated from acne skin and 53 from normal skin. For acne skin, 44 (74%) are classified in the subtype IA_1_, 7 (11%) in the subtype IA_2_, 2 (3%) in the subtype IB, 6 (10%) in the phylogenetic group II, and 0 in the phylogenetic group III. For normal skin, 27 (50%) are classified in the subtype IA_1_, 6 (11%) in the subtype IA_2_, 10 (18%) in the subtype IB, 7 (13%) in the phylogenetic group II, and 3 (6%) in the phylogenetic group III.

AD is a chronic inflammatory disease characterized by itchy skin, affecting children (10-20%) and adults (1−2%) in industrial countries [26,27]. There is evidence that human skin microbiome dysbiosis promotes AD [28]. Through sampling the skin microbiome of lesional and non-lesional skin regions of AD and healthy patients, authors showed that *Cutibacterium acnes* (*C. acnes*) abundance correlates inversely with *Staphylococcus aureus* (Figure 1E) [29,30]. The presence of this pathogen is known as the main factor that exacerbates AD and it has been suggested that antimicrobials from human skin commensal bacteria protect against *S. aureus* and are deficient in AD patients [31]. Rosacea is another dermatological disorder characterized by chronic inflammation of the face in adults, mostly women, after the age of 30 years [32]. Colonization of the skin by mites such as *Demodex* is a major pathogenic factor associated with rosacea skin conditions [33,34]. Interestingly, this mite has an associated microbiome, namely endosymbionts such as *Corynebacterium kroppenstedtii subsp. demodicis* [35], *Bacillus oleronius*, and *Bacillus cereus* [36], which are suggested to play a role in the pathogenicity of this disease [37]. However, the dysbiosis and the loss of relative abundance of *Cutibacterium acnes* has also been observed and correlated with the severity of the disease (Figure 1F) [38,39]. Psoriasis is also a chronic inflammatory disease characterized by hyperproliferation of keratinocytes and increased inflammation, affecting approximately 2% of the population [40]. Psoriasis is one of the most common immune-mediated skin disorders [41]. Alteration of microbial communities is observed within the bacterial populations of lesional and non-lesional skin regions of psoriasis versus healthy patients [42,43,44]. This strongly associates the dysbiosis with the progression of the disease [45,46], particularly with an imbalance of the dominant genera, Cutibacterium (decreased), Corynebacterium, and Staphylococcus (Figure 1G) [47,48]. Acne vulgaris is the most common dermatological condition worldwide [49]. Aggregating several risk factors (e.g., age, skin type, hormonal changes) and prevalent in the sebaceous/lipid-rich skin sites (e.g., face and back) [50,51], acne involves chronic inflammation of the pilosebaceous unit [52], often persisting into adulthood [53]. Extensively described and studied in past centuries [54], and firmly associated with the skin microbiome and *Cutibacterium acnes* proliferation [55,56], the paradigm of this dysbiosis is now changing. Recently, many authors observed that the relative abundance and bacterial load of *C. acnes* are not significantly different between acne and healthy patients (Figure 1H,I) [57,58,59,60,61,62]. In line with these findings, advances in sequencing technologies and in research in the past few decades have revealed the diversity of *C. acnes* at subspecies (*C. acnes* subsp. *acnes* (phylotype I), *C. acnes* subsp. *defendens* (phylotype II), *C. acne* subsp. *elongatum* (phylotype III)) and subtype levels (phylogenetic groups IA_1_, IA_2_, IB, IC, II, III) (Figure 2) [63]. This is responsible for the recent paradigm shift: rather than a sudden proliferation in the skin appendages, a decrease in the diversity between the six phylogenetic groups, rather than *C. acnes* proliferation, has been associated with acne progression (Figure 1J,K) [64,65].

For the four skin disorders succinctly described above, associated dysbiosis is correlated with a loss of Cutibacterium relative abundance and/or diversity at the genus and/or species levels. These alterations have a negative impact on skin biology by generating a loss of biological functions essential for skin homeostasis, possibly leading to inflammation. Therefore, in this review, two main questions are addressed: (1) How does Cutibacterium contribute to the physiology of healthy skin? and (2) How could probiotics better prevent and/or treat the dysbiosis disorders “on site” to restore the natural healthy functions of the skin?

## 2. Major Contributions of *Cutibacterium acnes* to Skin Homeostasis

The human microbiome plays a crucial role in human health. Different gut and skin diseases have been associated with microbiome dysbiosis, “elucidating by contraposition” its importance in the maintenance of a healthy state [69]. The gut microbiome is the most extensively studied human microbiome [70]. It has essential functions in the healthy state of the gut, such as protection against pathogen invasion, nourishing the host cells with their metabolic products, reinforcing the intestinal barrier, and training and modulating the immune system [71]. Since some skin diseases relate to dysbiosis of the natural microbiome, it is also expected that the skin microbiome plays a central role in skin homeostasis. One of the key players in skin homeostasis is *Cutibacterium acnes,* a Gram-positive rod bacterium that is aerotolerant anaerobic and does not produce spores [72]. It has coevolved with the host to reside in the pilosebaceous units, where oxygen and easily accessible nutrients are scarce [21]. To survive in the harsh and lipid-rich environment of the pilosebaceous units, *C. acnes* acquired genes to modulate and metabolize, inter alia, host skin lipids [73]. 

### 2.1. Lipid Modulation 

**Figure 3:**
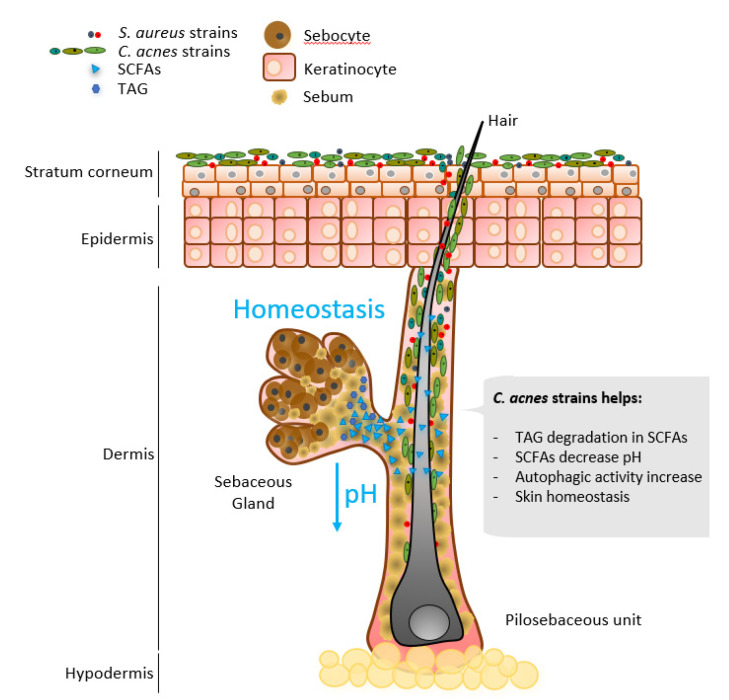
Selected *Cutibacterium acnes* major contributions in skin homeostasis.

To obtain energy from the abundant triacyclglycerols in sebum, *C. acnes* secretes a triacylglycerol lipase, GehA [74]. As a product of triacylglycerol fermentation, *C. acnes* secretes short-chain fatty acids (SCFAs) (Figure 3). The major SCFAs are acetate (C2), propionate (C3), and butyrate (C4) [75]. Less is known about the effect of SCFAs on the skin, although SCFAs metabolized by the gut microbiome have been shown to contribute to the maintenance of the colonic epithelium barrier by modulating epithelial tight junctions, colonocyte proliferation, final differentiation, and apoptosis [76].

As a result of its metabolism, *C. acnes* predominantly produces propionic acid, from which its former name, *Propionibacterium*, comes [21]. The role of propionic acid in the skin is yet to be uncovered, although it contributes to maintaining the acidic layer on the skin’s surface [77]. The physiological pH of healthy skin ranges from 4.1 to 5.8 [78]. Increased pH has been reported in AD, irritant contact dermatitis, ichthyosis, rosacea, and acne, but also in aged and dry skin [78]. Enzymes involved in maintaining skin barrier function, epidermal cell differentiation, and lipid production and accumulation are pH-dependent [78]. For example, acid sphingomyelinase hydrolyzes sphingolipids to liberate ceramides on the stratum corneum, an essential molecule on the skin [79]. Its activity is pH-regulated and AD has been correlated with impaired sphingomyelinase activity and high skin pH [80,81].

### 2.2. Follicular Niche Competition

**Figure 4:**
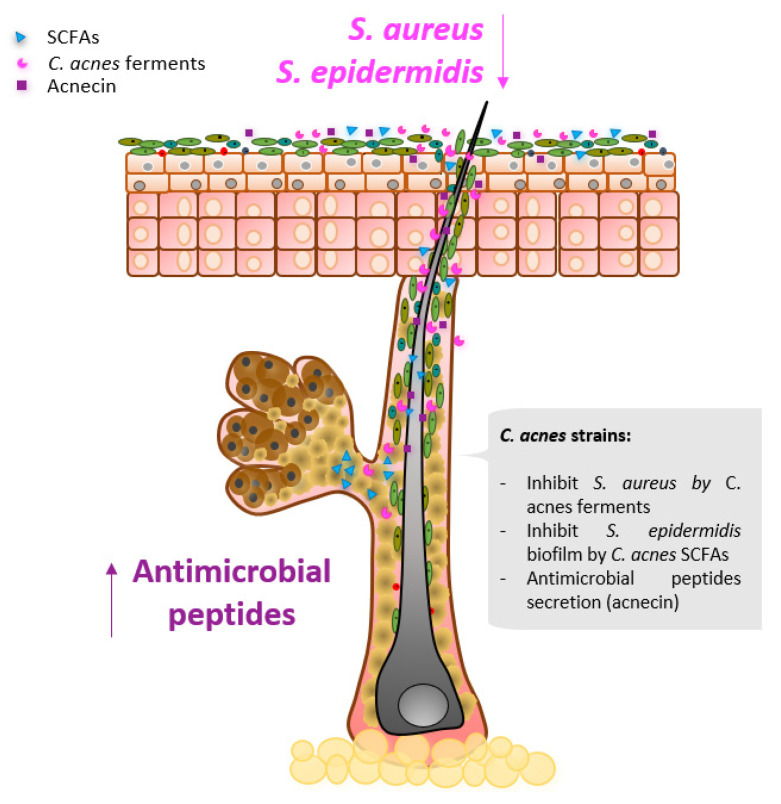
Selected *Cutibacterium acnes* major contributions in niche modulation.

Skin commensals are highly adapted to live in specific niches, in which they thrive and outcompete pathogens for nutrient acquisition [15]. Different strategies of niche modulation by *C. acnes* have been described in the literature (Figure 4). 

Recently, a biosynthetic gene (BSG) cluster capable of producing the antimicrobial thiopeptide cutimycin was identified in specific *C. acnes* strains from phylotypes IB and III [82]. Interestingly, only 8 of 219 screened *C. acnes* isolates contained the cutimycin BGC in their genome. Highlighting the selective niche competition of skin commensals, the expression levels of the BSG cluster producing cutimycin were increased when *C. acnes* was anaerobically cocultured with strains from the genus Staphylococcus, and BSG cluster expression levels were decreased when *C. acnes* was anaerobically cocultured with strains from the genus Corynebacterium [82]. Cutimycin was tested in vitro and was shown to possess antimicrobial activity against *Staphylococcus aureus*, but not Actinobacteria phyla. In vivo sampling showed a decreased ratio of Staphylococcus to *C. acnes* in individual hair follicles positive for cutimycin BCG compared to hair follicles where the cutimycin BSG cluster was not detected. 

*C. acnes* is the main colonizer of the pilosebaceous unit niche, where it produces the SCFAs propionic acid, isobutyric, and isovaleric acid in anaerobic conditions [83]. These SCFAs were shown to restore *S. epidermidis* antibiotic sensitivity by reducing its capacity to form biofilms [83]. *S. epidermidis* biofilm formation is related to skin disorders and rarely reported in healthy skin [84]. An older study investigated the antimicrobial activity of acnecin, a peptide produced by a subset of *C. acnes* strains. It was reported that acnecin inhibits the growth of other *C. acnes* strains that do not produce acnecin [85]. 

### 2.3. Immune Modulation 

Different skin-resident immune cells contribute to tissue homeostasis [2]. The major skin-resident immune cells are the myeloid and lymphoid cell subsets, which include a variety of specialized cell families that contribute to skin homeostasis, inflammation, and tissue reconstruction [86]. The immune cells on the skin participate in tight interactions with the skin microbiome to keep the skin healthy. It has been shown that germ-free mice had lower immune responses to pathogens, which were rescued by *S. epidermidis* recolonization on the skin [87]. Furthermore, *S. epidermidis* activates keratinocytes innate immune signaling pathways, triggering an increase in antimicrobial peptides (AMPs) targeting *S. aureus* [88]. Two different studies investigated immune interactions among *C. acnes* with keratinocytes and sebocytes. It was reported that *C. acnes* would not trigger an immune response unless environmental changes triggered higher production of SCFAs. These studies highlighted the immune tolerance of the skin towards *C. acnes* on homeostasis [89,90]. Another study explored the immune response of the skin to different *C. acnes* phylotypes, showing very different immune response patterns for acne and health-associated *C. acnes* phylotypes [91]. Finally, other research has described the enhanced autophagy activity of keratinocytes upon *C. acnes* interaction [92]. Autophagy is a major cellular defense mechanism to fight pathogen invasion [93]. It was suggested that low levels of hair follicle colonization by *C. acnes* could bring antimicrobial protection by locally enhancing autophagic activity in keratinocytes [92]. In the studies described above, it is reflected that in healthy skin, associated distribution of *C. acnes* phylotypes is tolerated and trains the host immune system. *C. acnes* has been described to promote the activation of T helper type 1 (Th1) cells in vivo [94]. Th1 cells belong to the cluster of differentiation 4 (CD4) Th group and are essential for intracellular pathogens eradication, such as *Listeria monocytogenes*. The other CD4 Th cells are type 2 (Th2) and are necessary for antibody production against extracellular organisms. The equilibrium of Th1/Th2 response is crucial for proper immune functioning [95]. Interestingly, AD is characterized by a shift in the Th1/Th2 equilibrium towards strong Th2 cytokine expression, triggering an allergic reaction on the skin [96]. In a study on mice with AD, *C. acnes* injections increased the Th1/Th2 ratio by enhancing the Th1 response. Th1 stimulation increased interleukin 12 (IL-12) and interferon gamma (INF-γ) expression, which have been described to counteract the effects of Th2 cells. Th1 stimulation in mice resulted in reduced development of AD compared to the control [97]. In another experiment performed on mice, using in vivo injections of *C. acnes* in malignant melanoma (MM), Th1 cells activated by *C. acnes* produced the antitumor cytokines IL-12, tumor necrosis factor alpha (TNF-α), and INF-γ [98]. Subcutaneous granuloma formation, which plays a major role in antitumor immunotherapy, was also observed. The tumor size in mice with malignant melanoma treated with *C. acnes* was significantly smaller than in the control mice [98]. This study concluded that intratumoral injection of *C. acnes* vaccine (ITPV) suppressed MM by inducing IL-12, TNF-α, and IFN-γ expression, along with granuloma formation.

### 2.4. Oxidative Stress Mitigation

**Figure 5:**
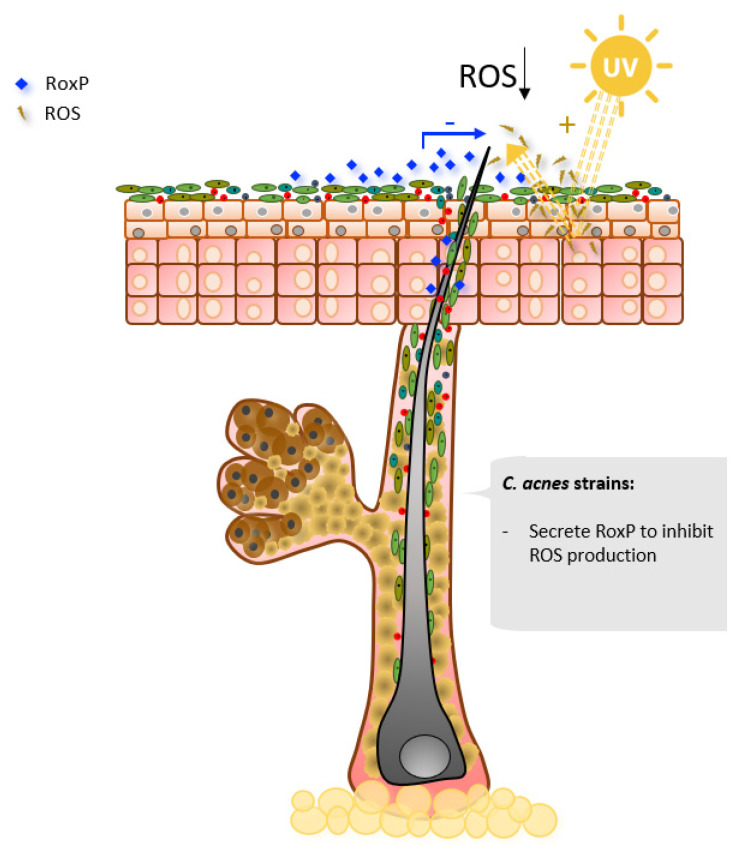
Selected *Cutibacterium acnes* major contributions in oxidative stress.

The skin is constantly exposed to UV radiation, which triggers the formation of reactive oxygen species (ROS) [99]. ROS oxidize lipids, proteins, and DNA, leading to cell damage and contributing to skin carcinogenesis [100]. Epithelial cells have multiple defense mechanisms to reduce ROS levels on the skin [101], such as the production of melanin and enzymes with antioxidant properties [102]. In addition to the ROS-mitigating strategies of epithelial cells, the most abundant secreted protein of *C. acnes*, radical oxygenase of *Propionibacterium acnes* (RoxP), has been reported to exert antioxidant activity (Figure 5) [74,103]. RoxP is the first extracellular bacterial antioxidant enzyme to be characterized [103]. RoxP is constitutively expressed and two variants with 83% amino acid identity have been described, one for *C. acnes* clade I and the other for clades II and III, both showing similar levels of antioxidant activity. RoxP was shown to increase the viability of ROS-stressed monocytes and keratinocytes in vitro, even to higher levels than the non-stressed group [104]. Furthermore, in actinic keratosis (AK), an initial stage of non-melanoma skin cancer, host cells antioxidant function is shown to be deficient [105]. In AK-affected sites, 16sRNA analysis showed a decrease in *C. acnes* populations, and RoxP levels detected in vivo by a capacity biosensor were lower compared to healthy areas [106,107].

## 3. How to Prevent and/or Treat “On Site” Dysbiosis Disorders

Dysbiosis of microbiome composition and function make microbiome-modulating strategies an interesting novel field of research for treatment of dysbiotic conditions. Currently, microbiome-modulating strategies have been mainly aimed at modulating the gut microbiota to redress dysbiotic patterns of the microbiome associated with disease [108]. Strategies aimed at modulating the gut microbiota involve using probiotics, prebiotics, and synbiotic and fecal microbiota transplants [109]. Microbiota transplantations are based on transferring the microbiome from a healthy subject to the dysbiotic receiver. Fecal microbiota transplants (FMT) have been proven to be effective for restoring the phylogenetic richness of the recipient’s intestinal microbiota, effectively treating gastric *Clostridium difficile* infections [110].

Microbiome modulation through the usage of probiotics, prebiotics, and synbiotics already has a long history of health claims through oral usage. Probiotics are defined as “live microorganisms which when administered in adequate amounts confer a health benefit on the host” [111]. Microorganism genera most commonly used are Lactobacillus, Bifidobacterium, Enterococcus, Lactococcus, Streptococcus, Bacillus, and yeast species such as *Saccharomyces boulardii* [112]. In light of the research conducted mainly in the last 10 years, the health benefits of these probiotics, administered through the gastrointestinal tract, act through four different mechanisms of action: (i) improvement of the epithelial barrier function, (ii) interference with pathogenic bacteria, (iii) immunomodulation, and (iv) influence on other organs of the body through the immune system [112]. Different strains of microbial species have specialized enzymatic activities and varied metabolic strategies, even within one species [113,114]. The mechanistic basis of probiotics is not associated with the genus or species of a microorganism, but claims can only be made for a few specially selected strains of a particular species [115]. Prebiotics are substrates selectively utilized by host microorganisms, conferring health benefits [116]. Such selectivity was shown for the gut commensal Bifidobacterium, which can be promoted by the ingestion of substances such as fructo-saccharides, oligosaccharides, and other prebiotics [117]. Synbiotics are products containing both prebiotics and probiotics. The implied synergism in the term reserves the definition “for products in which the prebiotic compound selectively favors the probiotic compound” [109]. Under this definition, a combination of an oligofructose and Bifidobacterium would classify as a synbiotic. Postbiotics refer to non-viable microorganisms (mainly heat-treated probiotic cells), lysed microbial cells, cell-free supernatants containing metabolites produced by probiotic strains, or purified key components from this supernatant [118]. These non-viable products have been tentatively termed postbiotics, but international consensus has yet to be reached [119]. The effects of non-viable microbial components such as heat-killed or UV-inactivated cells and cellular components when the cell is destroyed through lysis (DNA, RNA, proteins/peptides, polysaccharides, lipids, and others) have been shown to be immunomodulatory [120,121,122,123]. The metabolites derived from microbial metabolism include synthesized metabolites (SCFAs, bacteriocins, antioxidants, and others).

### 3.1. Skin Microbiome Modulation Strategies

Dysbiosis of the skin microbiome, associated with skin disorders, could be altered via multiple mechanisms: skin microbiome transplant, prebiotics, probiotics, synbiotics, and, putatively, postbiotics. Whole skin microbiome transplantation would require collecting the skin microbial community, which is challenging to achieve compared to fecal microbiota samples, as a culturing step is always required. Another ongoing challenge regards the uncultivability of microorganisms in vitro, also known as the “great plate count anomaly” [124]. Therefore, the performance of skin microbiome transplantation analogous to FMT is not scalable or industry applicable.

Recent years have seen a sharp increase in clinical investigations of probiotics and postbiotics used in dermatology. Processes to produce such non-viable fermentation products (postbiotics) are industrially scalable, can easily be formulated into products, and are widely used in the cosmetic industry. Probiotics, on the other hand, pose a challenge in terms of formulation and packaging to ensure the viability of the microorganism [125]. As a result, most suppliers formulate using postbiotics. Nevertheless, while postbiotics of skin commensals may protect against UV-induced oxidative damage, hyperpigmentation [126], and pathogens such as *S. aureus* [127], reintroducing viable microorganisms into their adapted niche has the potential to modulate the microbiome [128]. Skin microbiome modulation could mitigate or potentially eliminate pathological skin conditions, analogous to strategies in the gut. Callewaert et al. recently (2021) reviewed efforts undertaken in skin microbiome modulation strategies [128] and reintroduced the term bacteriotherapy to describe probiotics and postbiotics.

### 3.2. Bacteriotherapy in Dermatology

A recent review conducted in 2019 by Yu et al. investigated the interventional outcomes of oral probiotics and topical bacteriotherapy for dermatological conditions. Oral probiotics can be either consumed as encapsulated freeze-dried bacteria [129] or consumed through fermented foods such as yogurt, kefir, kimchi, and others [130]. In addition to alleviating gastrointestinal disorders, recent studies have investigated the usage of encapsulated probiotics in dermatology. It was noted that most clinical studies investigating probiotics in dermatology have investigated the effects of orally consumed probiotics for their interventional outcome, mainly aimed at alleviating acne and AD [131]. Although initial interventional outcomes of oral probiotics on dermatological conditions show promising results, we focus in this review on the underexplored potential of topical probiotics as a novel route for the prevention or treatment of “on site” dysbiosis disorders. Excluding duplicates from meta-analyses on oral and topical probiotics used in dermatology, only 9 out of 72 studies focused on topical bacteriotherapy. In addition to those reviewed by Yu et al., five studies were added that matched our search criteria on PubMed (“topical”, “probiotic”, “bacteriotherapy”, “dermatology”). Two were recent interventional studies investigating the outcome of topical bacteriotherapy in acne^24^ and AD^25^ and three additional studies [133,134,135] investigated the application of bacteriotherapy on healthy [132,133] and sensitive skin [134]. The first is a recent study by Karoglan et al. (2019) [135], who investigated the application of a mixture of probiotic strains (SLST types: C3, K8, A5, F4) from the skin commensal *C. acnes* in 14 patients with acne. Prior to topically applying the different mixtures of commensal probiotic strains, the skin microbial community was reduced using benzoyl peroxide to enhance the potential for transplantation [135]. As it has been noted that mixtures of strains from different phylogenetic lineages have synergistic effects on transplantation, two different mixtures of strains were assessed. The strains applied were selected based upon their production of linoleic isomerase, a biomarker putatively associated with inflammation [135]. This open-label study, without a control group, reported a significant reduction in total lesions compared to baseline. Interestingly, the applied probiotics could be detected after treatment in 50% of the treated patients, suggesting their ability to engraft onto the skin. No adverse events were reported by the patients, indicating that topical probiotics with *C. acnes* are safe. The second study included for revision is a randomized, placebo-controlled study conducted by Butler et al. (2020) [136], where the effect of the gut commensal *Lactobacillus Reuteri* (DSM 17938) strain was investigated in 36 adults with AD. When comparing the probiotic to the control, there was no significant improvement in scoring atopic dermatitis (SCORAD) index score. Thirdly, an interventional placebo-controlled study by Di Marzio et al. (2008) [132] was included, in which the authors investigated the application of non-viable commensal *Streptococcus thermophilus* cells on healthy skin of 20 elderly subjects. After receiving twice-daily dosages for 15 days, they reported a significant increase in hydration (*p* = 0.001), although no significant difference in transepidermal water loss (TEWL) was reported. The fourth study included is an interventional study by Nodake et al. (2015) [133] applying viable commensal *S. epidermis* strains to the healthy skin of 21 elderly subjects. They received twice-weekly dosages for 4 weeks in a double-blinded, placebo-controlled study. A significant reduction in TEWL (*p* < 0.05), significant increase in lipid content (*p* < 0.05), and significant increase in hydration (*p* < 0.05) were observed. Furthermore, they reported a significant reduction in the pH of the skin of elderly participants (*p* < 0.05). Interestingly, a meta-analysis of 63 randomized controlled trials revealed that the use of products with a low pH (4) is currently the most effective strategy for improving the skin barrier [137]. The fifth additional study is a randomized, placebo-controlled study by Gueniche et al. (2010) [134]. They applied lysed *Bifidobacterium longum reuteri* postbiotics to participants with sensitive skin twice daily for 2 months. The treatment group showed significantly decreased skin sensitivity as assessed with a lactic acid test (*p* < 0.01). Furthermore, they reported significantly improved resistance to physical aggression in the form of tape-stripping (*p* < 0.01). With the addition of these five recent studies, a total of 14 studies investigating the interventional outcomes of topical probiotics and postbiotics were reviewed. All interventional studies are summarized in Supplementary Table S1, including strain origin, viability, dose, and main results. Furthermore, bacterial phages naturally present on the skin microbiome are being studied and investigated for their microbiome-modulating properties by targeting specific species such as *C. acnes* [138].

### 3.3. Probiotics Used in Dermatology

The use of probiotics in dermatology involves mainly oral probiotics (80%), and of the 14 studies included in this review, only eight investigated viable bacterial probiotics. Only 6 out of 14 studies investigated interventional outcomes of skin commensal strains, as the other studies included strains isolated from the gastrointestinal tract (*Lactobacillus reuteri DSM 17938, Lactobacillus plantarum, Bifidobacterium longum reuteri*)*,* urogenital tract (*Lactobacillus johnsonii*), thermal water (*Vitreoscilla filiformis*), and the skin of Amerindian peoples (*Nitrosomonas eutropha*). Despite the promising results of the clinical investigations presented in this review (mainly targeted towards acne and AD), research on topical probiotics is still in its very early stages. There is an underexploited opportunity to apply viable commensal strains that have the potential to restore the dysbiosis of skin disorders. There is an unmet need for patients to find effective treatments with limited side-effects. Commensal skin probiotics should be further explored to provide patients with novel treatment strategies with strain-associated beneficial properties that are adapted to the environmental habitat of application.

### 3.4. Safety Functionality and Technical Feasibility for Skin Probiotics

As topical probiotics are nascent in the cosmetic and dermatological industry, their regulatory classification is unclear and may fall between cosmetics, medical devices, and pharmaceuticals [139]. Rigorous standards for the selection of specific strains according to their safety, functionality, and technical feasibility have been established for gut probiotics through years of research and development [140]. A review published in 2017 outlined the safety, functional, and technological aspects that must be considered when selecting probiotic strains for usage in the gastrointestinal tract. These were compiled from recommendations by the Food and Agricultural Organization, World Health Organization, and European Food Safety Authority [140]. Adapting similar rigorous selection criteria for skin probiotic strains would be desirable for skin probiotics, to protect and deliver value to patients, as depicted in Table 1 using probiotics with *C. acnes* as an example. 

## 4. Conclusions

*C. acnes* is a commensal bacterium of human skin. As reviewed in this paper, the relative abundance and/or diversity of clonal population is associated with skin health and is involved in maintaining the essential biophysiological functions of the skin. The history of this genus is constantly evolving as research and technology progress. Mainly regarded as the cause of disease due to the virulence and pathogenic factors associated with a large part of its clonal population, the paradigm of *C. acnes* colonization is now shifting. From a uniquely opportunistic pathogen, the nascent alternative vision encourages the potential use of some specific strains of *C. acnes* as efficient probiotics to restore the natural equilibrium of a disbalanced skin microbiome.

## Figures and Tables

**Table 1:** Selection criteria for skin probiotic strains (adapted from and Katarzyna Śliżewska [140]).

**Safety**	**Statement**
**Human or animal origin**	The novel genus Cutibacterium contains the human cutaneous species formerly known as *Propionibacterium acnes*, *Propionibacterium avidum, Propionibacterium granulosum* [21].
**Isolated from the skin** **of healthy individuals**	*C. acnes* is a prevalent bacterial commensals on healthy skin [19]. Studies investigating probiotic *C. acnes* strains used isolates from the skin of healthy individuals [135,141].
**History of safe use**	*C. acnes* has no long history of use yet, see “no adverse effects” for safety indication [135].
**Precise diagnostic identification** **(phenotype and genotype traits)**	SLST analysis allows the strain-level identification of *C. acnes* species [142].
**Absence of data regarding an** **association with infective disease**	Strains of *C. acnes* have been reported as opportunistic pathogens. Virulence is related to certain phylotypes or strains (e.g., phylotype IA has been associated with acne [143]).
**No adverse effects**	No adverse events were reported in two studies applying *C. acnes* probiotics [135,141].
**Absence of genes responsible for antibiotic** **resistance localized in non-stable elements**	No detectable differences in the MIC of 21 antibiotics were observed between parent strains and their plasmid-cured derivatives for *C. acnes* [144].
**Functionality**	**Statement**
**Competitiveness with respect to the microbiota inhabiting the ecosystem of the skin**	Antagonistic activity between *S. epidermis* and *C. acnes* strains [145].
**Ability to survive and maintain the metabolic activity and to grow in the target site**	Applied probiotic strains of *C. acnes* could be detected well beyond application days, indicating their ability to survive in the target site [141].
**Resistance to skin salts and enzymes**	*C. acnes* is adapted to lipid-rich anaerobic environment of sebaceous follicles [146].
**Resistance to low pH of the skin**	*C. acnes* can compensate for the low pH by utilizing arginine deiminase [147].
**Competitiveness with respect to microbial species inhabiting the ecosystem of the skin (including closely related species)**	*C. acnes* inhibits biofilm formation of *S. epidermidis* [83]. *C. acnes* produces an antibiotic that targets specific *C. acnes* strains [85].
**Antagonistic activity towards pathogens (e.g., *S. aureus)***	*C. acnes* produces an antibiotic against *S. aureus* and *C. acnes* ferments have been shown to inhibit methicillin-resistant *S. aureus* [82,127]. *S. epidermis* degrades proteins associated with *S. aureus* biofilm formation [148]. *S. lugdunensis* inhibits growth of *S. aureus* through production of AMP lugdunin [149].
**Resistance to bacteriocins and acids produced by the endogenic skin microbiota**	*C. acnes* and *S. epidermidis* coexist in the skin as stable heterogeneous communities of strains [19].
**Adherence and ability to colonize some particular sites within the host organism,** **and an appropriate survival rate on the skin**	Different body sites are shown to be colonized by different multi-phyletic communities of *C. acnes* [15].
**Technological feasibility**	**Statement**
**Easy production of large amounts of biomass and high productivity of cultures**	Steady-state continuous culture of *Propionibacterium acnes* was achieved [150].
**Viability and stability of the desired properties of probiotic bacteria during the fixing process (freezing, freeze-drying), preparation, and distribution of probiotic products**	Not yet established.
**High storage survival rate in finished products (in aerobic and micro-aerophilic conditions)**	*C. acnes* probiotic solution was stable for at least 1.5 months at room temperature [141]
**Guarantee of desired sensory properties of finished products (in the case of the cosmetics industry)**	Not yet established
**Genetic stability**	The sequenced strain exhibited 100% identity on the 16S ribosomal RNA to several isolated *C. acnes* [73].
**Resistance to bacteriophages**	*C. acnes* phylotypes show selective resistance or sensitivity to bacteriophages [151].

## Supplementary Materials

The following are available online at https://www.mdpi.com/2076-2607/9/3/628/s1.
