# Supplementary material for: From Dysbiosis to Healthy Skin: Major Contributions of Cutibacterium acnes to Skin Homeostasis
Source: Microorganisms. 2021 Mar 18;9(3):628. doi: 10.3390/microorganisms9030628 (PMC8003110; doi:10.3390/microorganisms9030628)
Supplement: Supplementary file 1 [file microorganisms-09-00628-s001.pdf]

**Supplementary table 1.** Interventional studies with topical bacteriotherapy in dermatology.

| Target | Source | Participants | Probiotic Strains | Strain origin | Skin commensal | Downstream process | Viable | Dose | Main results |
|---|---|---|---|---|---|---|---|---|---|
| Atopic dermatitis | Nakatsuji *et al,* 2018[1] | 14 Adults | *Staphylococcus epidermidis, Staphylococcus hominis, Staphylococcus capitis, Staphylococcus warneri* | Skin | YES | freeze-dried | YES | Single dose $1 \times 10^5$ CFU/cm2 | After 1 week ">90% reduction in *S. aureus* load ($p = 0.013$) and significant improvements in local EASI ($p = 0.029$)." |
| Atopic dermatitis | Myles et al, 2018[2] | 10 Adults | *Roseomonas mucosa* | Skin | YES | freeze-dried | YES | Twice weekly for 6 weeks (increasing dosages $4 \times 10^3$, $4 \times 10^4$, $4 \times 10^5$) | "Significant reduction in pruritus ($p < 0.01$), SCORAD values, and steroid usage ($p < 0.05$)." |
| Atopic dermatitis | Blanchet-Rethore et al, 2017[3] | 21 Adults | *Lactobacillus johnsonii* | Vagina | NO | heat inactivated | NO | Twice daily for 21 days days | "Reduction in *S. aureus* load, correlating with $4.93 \pm 8.23$ decrease in mean SCORAD between days 8 to 28 ($p = 0.012$)." |
| Atopic dermatitis | Di Marzio et al, 2003[4] | 11 Adults | *Streptococcus thermophilus* | Skin, plant and dairy | NO | sonicated | NO | Twice daily for 14 days | Significant improvement "in scores of erythema, pruritus, vesiculation, and scaling ($p = 0.003$). Stratum corneum ceramide levels increased ($12.86 \pm 1.97$pmol/cm$^2$; $p = 0.002$)." |
| Atopic dermatitis | Gueniche et al, 2008[5] | 75 Adults and Children | *Vitreoscilla filiformis* | Thermal water | NO | lysate | NO | Once daily for 30 days | "Significant reduction of SCORAD values ($p = 0.0044$), pruritus ($p = 0.0171$), loss of sleep ($p = 0.0074$)" |

| | | | | | | | | | |
|---|---|---|---|---|---|---|---|---|---|
| Atopic dermatitits | Butler, 2020[6] | 36 adults | *Lactobacillus reuteri (DSM 17938)* | Gastrointestinal | NO | freeze-dried | YES | Twice daily for 8 weeks ($10^8$ CFU/gram) | Both vehicle and verum compared to baseline improved signifcantly (SCORAD index). No significant difference in the overall effect between the control and treatment |
| Acne vulgaris | AOBiome Clinical trial (2018) (not published *) | 358 Adults | *Nitrosomonas eutropha* | Amerindian skin (not Western skin) | NO | NA | YES | NA | "2-point reduction in Investigator's Global Assessment, ($p$ = 0.03) at 12 weeks. Number of inflammatory lesions also reduced ($p$ = 0.028). " (results from AOBiome press-release) |
| Acne vulgaris | Karoglan et al, 2019[7] | 14 Subjects | *Cutibacterium acnes (C3, K8, A5, F4)* | Skin | YES | NA | YES | Twice daily for 5 weeks ($10^6$ CFU/g) | Significant reduction in non-inflammed lesions (C3 + K8 p = 0.029, C3 + K8 + A5 + F4, p = 0.036) |
| Seborrheic dermatitis | Geuniche et al, 2008[8] | 60 Adults | *Vitreoscilla filiformis* | Thermal water | NO | NA | NO | Once daily for 4 weeks | "Significant reduction in erythema and scaling treated vs vehicle (Chi-square, P < 0.0001)" |
| Non-Healing Ulcers | Peral et al, 2010[9] | 34 diabetic and non-diabetic adults | *Lactobacillus plantarum* | Gastrointestinal | NO | NA | YES | Daily for 10 days (whole culture 10 5 / ml) | "10 days of treatment resulted in a 3-fold decrease in biofilm producing bacteria ($p$ < 0.001). After 30 days, 43% of diabetics and 50% of non-diabetics experienced ulcer resolution." |
| Burns | Peral et al, 2009[10] | 80 Adults | *Lactobacillus plantarum* | Gastrointestinal | NO | NA | YES | Daily for 10 days (whole culture $10^5$ / ml) | "7-13 days of treatment increased rates of granulation tissue (relative rate = +17.19) compared to the standard of care for delayed 3rd degree burns. No significant |

| | | | | | | | | | |
|---|---|---|---|---|---|---|---|---|---|
| | | | | | | | | | difference observed in 2nd degree or early 3rd degree burns.” |
| Sensitive skin | Gueniche, 2010[11] | 66 Adults | *Bifidobacterium longum reuteri* | Gastrointestinal | NO | lysate | NO | Twice daily for 2 months | Significant decrease in skin sensitivity ($p < 0.01$), significant improved resistance to physical aggression ($p < 0.01$) |
| Healthy | Di Marzio, 2008[12] | 20 elderly | *Streptococcus thermop hilus* | Skin, plant and dairy | NO | sonicated | NO | Twice daily for 15 days | Significant increase in skin hydration ( $p = 0.001$) |
| Healthy | Nodake, 2015[13] | 21 adults | *Staphylococcus epidermis* | Skin | YES | freeze-dried | YES | Twice weekly for 4 weeks ($1.36 \times 10^9$ cells/mL) | Significant increase in hydration , significant decrease in TEWL ($p < 0.05$), significant increase in lipid content ($p < 0.05$), signifcant decrease in pH ($p < 0.05$) |

1. Nakatsuji, T. *et al.* Clinical improvement in atopic dermatitis following autologous application of microbiome therapy targeting Staphylococcus aureus. *Journal of Investigative Dermatology* **138**, S72 (2018).
2. Myles, I. A. *et al.* First-in-human topical microbiome transplantation with Roseomonas mucosa for atopic dermatitis. https://insight.jci.org/articles/view/120608/figure/1 (2018) doi:10.1172/jci.insight.120608.
3. Blanchet-Réthoré, S. *et al.* Effect of a lotion containing the heat-treated probiotic strain Lactobacillus johnsonii NCC 533 on Staphylococcus aureus colonization in atopic dermatitis. *Clin Cosmet Investig Dermatol* **10**, 249–257 (2017).
4. Marzio, L. D. *et al.* Effect of the lactic acid bacterium Streptococcus thermophilus on stratum corneum ceramide levels and signs and symptoms of atopic dermatitis patients. *Experimental Dermatology* **12**, 615–620 (2003).
5. Gueniche, A. *et al.* Effects of nonpathogenic gram-negative bacterium Vitreoscilla filiformis lysate on atopic dermatitis: a prospective, randomized, double-blind, placebo-controlled clinical study. *British Journal of Dermatology* **159**, 1357–1363 (2008).
6. Butler, É., Lundqvist, C. & Axelsson, J. Lactobacillus reuteri DSM 17938 as a Novel Topical Cosmetic Ingredient: A Proof of Concept Clinical Study in Adults with Atopic Dermatitis. *Microorganisms* **8**, 1026 (2020).
7. Karoglan, A. *et al.* Safety and Efficacy of Topically Applied Selected Cutibacterium acnes Strains over Five Weeks in Patients with Acne Vulgaris: An Open-label, Pilot Study. *Acta Dermato-Venereologica* **99**, 1253–1257 (2019).
8. Guéniche, A. *et al.* Vitreoscilla filiformis biomass improves seborrheic dermatitis. *J Eur Acad Dermatol Venereol* **22**, 1014–1015 (2008).
9. Peral, M. C., Rachid, M. M., Gobbato, N. M., Martinez, M. A. H. & Valdez, J. C. Interleukin-8 production by polymorphonuclear leukocytes from patients with chronic infected leg ulcers treated with Lactobacillus plantarum. *Clinical Microbiology and Infection* **16**, 281–286 (2010).

10. Peral, M. C., Martinez, M. A. H. & Valdez, J. C. Bacteriotherapy with Lactobacillus plantarum in burns. *International Wound Journal* **6**, 73–81 (2009).

11. Guéniche, A. *et al.* Bifidobacterium longum lysate, a new ingredient for reactive skin. *Experimental Dermatology* **19**, e1–e8 (2010).

12. Dimarzio, L. *et al.* Increase of Skin-Ceramide Levels in Aged Subjects following a Short-Term Topical Application of Bacterial Sphingomyelinase from Streptococcus Thermophilus. *Int J Immunopathol Pharmacol* **21**, 137–143 (2008).

13. Nodake, Y. *et al.* Pilot study on novel skin care method by augmentation with Staphylococcus epidermidis, an autologous skin microbe – A blinded randomized clinical trial. *Journal of Dermatological Science* **79**, 119–126 (2015).
